# Toleration and prejudice‐reduction: Two ways of improving intergroup relations

**DOI:** 10.1002/ejsp.2624

**Published:** 2019-09-19

**Authors:** Maykel Verkuyten, Kumar Yogeeswaran, Levi Adelman

**Affiliations:** ^1^ Faculty of Social and Behavioural Sciences Ercomer Utrecht University Utrecht The Netherlands; ^2^ University of Canterbury Christchurch New Zealand

**Keywords:** diversity, intergroup relations, prejudice reduction, tolerance, toleration

## Abstract

While a large body of social psychological research has shed light on the nature of prejudice and how to reduce it, we argue that such work does not address situations of cultural or religious outgroup beliefs and practices that are considered incompatible with one's own. The present theoretical article contrasts a prejudice‐reduction approach with a toleration‐based approach to consider the differences each have with regard to the attitude object they focus upon, the perceived reasonableness of the attitude, and the behavioral consequences each may lead to. In doing so, we consider the psychological processes involved in whether the negative attitude leads to negative actions. We conclude by arguing that a toleration‐based approach forms an important addition to the psychological thinking about cultural diversity and intergroup relations. Collectively, the present work makes a novel contribution to the social psychological literature by stimulating theory development and raising novel questions for empirical research.

## 1 INTRODUCTION

Although there have been general improvements in intergroup outcomes over the last few decades in several countries (e.g., Krysan & Moberg, 2016; Schafer & Shaw, 2009; Schuman, Steeh, Bobo, & Krysan, 1997; Welch & Sigelman, 2011), there is still significant debate over the acceptance and integration of different cultural groups into our increasingly diverse nations (Plaut, 2010; Verkuyten, 2014). Despite a large and valuable social psychological literature on the nature of prejudice, the psychological processes underlying prejudicial attitudes, and ways to try to reduce prejudice and discrimination (e.g., Beelman & Heinemann, 2014; Paluck & Green, 2009; Tropp & Mallett, 2011), prejudice reduction tactics have not yet fulfilled their potential of fully addressing intergroup conflict over pressing social issues (e.g., Dixon, Levine, Reicher, & Durrheim, 2012; Kalev, Dobbin, & Kelly, 2006; Lai et al., 2014; Paluck & Green, 2009). While much of the gap between the aims and results of prejudice‐reduction research may be explained by social structural constraints and the difficulty of applying complex interventions that can overcome the pervasive prejudicial messages that people have received over their life‐spans, we propose that there may also be tool–task discrepancies that limit our ability to address some of the sources of conflict in pluralistic societies. Specifically, intergroup conflicts that emerge from reasonable disapproval of outgroup beliefs, practices, and ways of living may not be successfully addressed by prejudice‐reduction approaches, and such strategies may even be counter‐productive if used in these cases. Our central argument is that in the aim of better understanding and managing the complexities of diversity, the social psychology of cultural diversity and intergroup relations should not be reduced to a psychology of antipathy and prejudice‐reduction, but also requires an understanding of disapproval and toleration of outgroup beliefs, practices, and ways of living.

In the present article, we aim to elucidate the key theoretical differences between what we will broadly call the prejudice‐reduction approach and the toleration‐based approach to managing the challenges of cultural diversity in order to provide a better understanding of both approaches’ potentials and limitations for improving intergroup relations.1Here, we will specifically focus on differences emerging from cultural, religious, or ideological diversity. Subsequently we will consider the psychological processes involved in each of these approaches and discuss the implications for prejudice‐reduction and toleration‐based initiatives. In doing so, we will explain why prejudice‐reduction strategies may be insufficient or inappropriate to addressing questions of intolerance and reasonable disapproval before highlighting the importance of developing a toleration‐based approach to intergroup relations. We conclude by identifying future directions for theoretical and empirical development within this new area.

### 1.1 Understanding (In)Tolerance

Tolerance is a buzzword across a wide range of settings and across a diverse ideological and political field for establishing multicultural justice and peaceful coexistence (Brown, 2006). The United Nations (2017) and the European Council (2008) promote tolerance, several nation‐states adopt policies to encourage tolerance, religious groups, civic associations, and various institutions promulgate tolerance, and schools teach tolerance to their students (Verkuyten, Yogeeswaran, & Adelman, 2019). Some of these initiatives equate tolerance with openness, being well disposed toward cultural others, or having a generalized positive attitude toward them. This is similar to social psychologists equating the adjective “tolerance” with being non‐judgmental and open to differences (“tolerant personality”) and using the term to describe a “tendency to be generally free of prejudice” (Duckitt, 1992, p. 8) or to have a positive outgroup attitude (Brewer & Pierce, 2005; Mummendey & Wenzel, 1999). On this understanding, tolerance is the opposite of prejudice and intolerance is equated with prejudice as a generalized negative feeling, belief, and behavior toward a group or an individual member of that group (e.g., Brandt, Chambers, Crawford, Wetherell, & Reyna, 2015).

However, this usage deprives the term of its principal distinguishing feature, which is that we tolerate what we disapprove of. There is a great deal of consensus about this classical meaning in the philosophical and political science literature (e.g., Cohen, 2004; Forst, 2013; Gibson, 2006; Horton, 1996; Nelson, Clawson, & Oxley, 1997; Walzer, 1997). The combination of a negative attitude with forbearance is what makes toleration psychologically distinctive and worth investigating in social psychology (Verkuyten & Yogeeswaran, 2017). Toleration requires one to put up with differences one disapproves of, such as religious and ideological beliefs, cultural practices, sexual orientations, and modes of behavior differing from one's own. Tolerance is not ignorance, indifference, apathy, or cultural relativism with an abstention of judgment, but presupposes a negative attitude toward outgroup beliefs and practices, together with intentional self‐restraint (i.e., not based on fear or compelled) from acting upon this attitude. Toleration “is the deliberate decision to refrain from prohibiting, hindering or otherwise coercively interfering with conduct of which one disapproves, although one has the power to do so” (Horton, 1996, p. 29). Like discrimination, tolerance typically implies a difference in power in which one is, or believes to be, in a position to be able to interfere with the behavior of the tolerated, but refrains from doing so.

The importance of studying (in)tolerance in addition to prejudice is further highlighted by research demonstrating that prejudice and intolerance can be weakly, or not, related (e.g., Crawford, 2014; Gibson, 2006; Klein & Zick, 2013; McIntosh, Iver, Abele, & Nolle, 1995; Van der Noll, Verkuyten, & Poppe, 2010; Wirtz, Van der Pligt, & Doosje, 2016). Furthermore, political science research examines tolerance in terms of being willing to grant the full rights of citizenship to groups one dislikes (Gibson, 2006; Mondak & Sanders, 2003). Additionally, research on *protected values* (Baron & Spranca, 1997), the *value protection model of justice reasoning* (Skitka, 2002; Skitka & Mullen, 2002), the *sacred value protection model* (Tetlock, 2003), the *world view defence model* (Brandt, Reyna, Chambers, Crawford, & Wetherell, 2014; Brandt & Van Tongeren, 2017), and *moral convictions* (Ellemers, 2017; Haidt, 2012) demonstrates that not all cultural and religious beliefs and practices can exist comfortably side‐by‐side. It is highly unlikely that group members who hold a strong conviction, be it cultural, ideological, or religious, will come to accept and approve of beliefs and practices of outgroup members who strongly subscribe to an alternative worldview. It is hard to value and celebrate diversity when one believes that certain forms of relations are unjust (gender inequality), certain forms of sexual behavior morally wrong (homosexuality), certain practices interfere with the rights and liberties of others (free choice of a partner, and the right to apostasy), and certain ideologies are oppressive (patriarchy). Because of their propositional content, all religions and cultures cannot be considered to have equal value. What makes a cultural, religious, or ideological belief critical and psychologically meaningful is that it is taken to be true (Crane, 2017). Devout believers, for instance, cannot be expected to accept the equal value of other belief systems (Tate, 2016).

Different beliefs and worldviews about what is right or wrong, true and false, cannot all be simultaneously and equally confirmed, but they can be tolerated. It is possible to disapprove of particular beliefs and practices, while accepting that other people should be able to fashion their life according to their desires and needs. Experimental research has demonstrated that making tolerance a salient aspect of national identity leads to stronger support for Muslim minority rights (Smeekes, Verkuyten, & Poppe, 2012). Similarly, mortality salience does not lead to negative reactions to an anti‐U.S. message when the value of tolerance is made salient (Greenberg, Simon, Pyszczynski, Solomon, & Chatel, 1992). Intergroup toleration can promote social cohesion and intergroup harmony despite concrete and potentially irreconcilable differences. The importance of toleration is that it allows people to accept that others have the right to live according to their own beliefs and values even when they are in conflict with our own. Tolerance does not require that one gives up or dilutes one's own beliefs and values and thereby is one of “the few viable solutions to the tensions and conflict brought about by multiculturalism and political heterogeneity” (Gibson, 2006, p. 21). In the words of former US President Kennedy, “Tolerance implies no lack of commitment to one's own beliefs. Rather it condemns the oppression or persecution of others”.

## 2 PREJUDICE AND DISAPPROVAL

There is a very large social psychological literature on prejudice in which a range of definitions are presented and discussed (see Brown, 2010; Duckitt, 1992). This literature tends to consider sexism, racism, Islamophobia, homophobia, and the like as special cases of the more general phenomenon of prejudice (Brown, 2010). Racism in particular has become the prototypical example of prejudice.2For example, Duckitt's (1992) extensive overview of research on “The social psychology of prejudice” is predominantly concerned with race prejudice (see also Jones, 1997). Additionally, although being prejudiced is typically equated with having a negative attitude, some modern manifestations of prejudice involve a blend of positive and negative aspects, with positive attitudes also having unfavorable consequences for disadvantaged groups, such as with benevolent sexism (Glick & Fiske, 2001). Indeed, some perspectives on prejudice and discrimination assert that prejudice can emerge not from antipathy, but rather from perceived incongruity of a person's social role with the stereotypes associated with their group (Eagly & Karau, 2002). Furthermore, there are different theoretical perspectives and models for studying prejudice ranging from implicit mental processes to social structural conditions (see Dixon & Levine, 2012).

These different aspects, distinctions, and perspectives make the field of prejudice research quite broad and difficult to evaluate. However, several common components of prejudice allow us to identify some key conceptual differences between the features of prejudice and the disapproval underlying (in)tolerance. This is important because thinking theoretically requires conceptual distinctions that make it possible to examine empirically the nature of prejudice and (in)tolerance, and when and why both might dissolve and bleed into one another in everyday life. In the empirical world, prejudice and (in)tolerance can be relatively independent but might also become closely connected and hard to distinguish. However, such observations are difficult to make without at least some idea about what prejudice and disapproval‐based (in)tolerance are all about. Developing the conceptual distinction between these phenomena gives us the tools to study the nature of these two processes, and to better understand how and when the two exist separately or coexist.

For our present purposes, we will discuss three aspects of prejudice and disapproval that can be used for making an analytical distinction between these two concepts. These relate to (a) the object of the attitude, (b) attitude reasonableness, and (c) the resulting behavior.

### 2.1 Attitude object

One difference between prejudice and disapproval lies in the object of the attitude. A key feature of prejudice is that “it is a social orientation towards whole groups of people or towards individuals because of their membership in a particular group” (Brown, 2010, p. 4). Thus, prejudice is commonly seen as a form of antipathy or hatred toward a group, such as African Americans, Mexicans, Muslims, Jews, or sexual minorities. There are many possible reasons for the group‐based antipathy, but what is shared is the focus on a category of people *as* people. Some research on prejudice is concerned with how social categorization processes that distinguish “us” and “them” provide a necessary condition for intergroup bias in which one's own group is favored over others (Diehl, 1990; Tajfel & Turner, 1979). Social categorization is, thereby, considered a key factor in intergroup animosity with stronger group differences often leading to greater intergroup bias (but see Park & Judd, 2005).

In contrast, the disapproval of intergroup tolerance is typically not applied to social categories and groups *per se* (Crick, 1971; Mendus, 1989; Mouritsen & Olsen, 2013). A categorical distinction between ingroup (“us”) and outgroup (“them”) does not make the outgroup (“them”), by itself, a subject for tolerance. Intergroup bias resulting from social categorization is not thought of as being redressable by tolerance. Furthermore, it makes little sense to say that one must dislike or hate a cultural, religious, or racial group of people to be tolerant of them. This would imply that one has to be a bigot in order to have the possibility of being tolerant and that a more racist person showing self‐restraint is more tolerant and virtuous (Forst, 2013; Horton, 1996).The result would be that prejudice and racism are turned into acceptable moral positions.

However, groups become the proper focus of disapproval‐based tolerance if accompanied by a defining set of values and beliefs and a community of practitioners.3This conceptualization differs from how political tolerance is typically studied in political science (Gibson, 2006). Political tolerance is about how far one is willing to grant equal rights to disliked groups, which differs from intergroup toleration (Vogt, 1997). Furthermore, with political tolerance it is often difficult to make a distinction between the fact that people can object to a particular practice of a group because they dislike the group or because they disapprove of the practice itself (Hurwitz & Mondak, 2002). An example is opinion‐based groups (being pro or anti an issue) that involve a social identity based on shared opinion with the related behavior (Bliuc, McGarty, Reynolds, & Muntele, 2007). And when ethnic, racial, and religious differences are reified and essentialized, the question of tolerating ethnic, racial, and religious groups arises. But in principle, the disapproval involved in (in)tolerance is not focused on social categories, but rather on specific dissenting beliefs and practices. It is around concrete issues (e.g., dress‐code, religious education, language use, dietary requirements, sexual practices, mosque building, parenting approaches) that ways of life collide and the need for toleration arises.

However, these issues contain two pieces of information: the group involved in the practice (e.g., Muslims) and the nature of the practice itself (e.g., dress‐code). Muslim immigrants founding an Islamic school differs from Christians founding a school, which in turn differs from Muslim immigrants wearing a headscarf. People either can respond in a prejudicial way to the group (“I dislike Muslims”) or disapprove of the particular practice (“I am against all religious education”), or a combination of the two. Furthermore, one can be intolerant of specific beliefs and practices of individuals or groups toward whom one has no negative feelings. Like parents who find certain practices of their children unacceptable (e.g., smoking), one can reject a specific practice (e.g., ritual slaughter of animals) of a group (Jews, Muslims) to whom one has neutral or even positive feelings (Hurwitz & Mondak, 2002; Sniderman, Tetlock, Glaser, Green, & Hout, 1989). Further, people can wish to deny forms of freedom of expression (e.g., burning of the national flag, forms of hate speech) to fellow ingroup members.

In another example, one can like Muslims as a group, but that does not have to mean that one approves of all their religious beliefs and practices. Research in the Netherlands has shown that those who object to what they consider unequal treatment of women and authoritarian childrearing practices among Muslim minorities do not necessarily show dislike or antipathy toward Muslims as a group (Hagendoorn & Poppe, 2012; Sniderman & Hagendoorn, 2007; see also Adelman & Verkuyten, 2019). Similarly, a large‐scale study in Canada found that a majority of people supporting the banning of religious symbols in the public sphere did not have anti‐Muslim sentiments (Breton & Eady, 2015; see also Bilodeau, Turgeon, White, & Henderson, 2018). And among national samples in the UK, France, Germany, and the Netherlands, a substantial portion of people with a positive attitude toward Muslims supported a ban on headscarves (Van der Noll, 2010). Similarly, analyzing data from six European countries, Helbling (2014) found that people in Western Europe make a distinction between their attitudes toward Muslims as a group and their attitudes toward the Muslim headscarf. And in an experiment in the context of the UK it was found that citizens’ uneasiness with Muslim immigrants is not the result of negative attitudes toward Muslims as a group but rather based on a rejection of specific religious behaviors (Helbling & Traunmüller, 2018). Collectively, these findings illustrate how people's attitudes toward an outgroup can be distinct from their disapproval of specific outgroup practices. Simply equating support for banning headscarves or objecting to the building of minarets with anti‐Muslim prejudice is not the whole story: “opposition to diversity is not simply a case of dislike of other groups. Often values come into conflict” (Jones & Dovidio, 2018, p. 43).

### 2.2 Attitude reasonableness

While both prejudice and disapproval involve negative attitudes toward a target object (i.e., toward an outgroup in the case of prejudice, or an outgroup belief, practice, or way of life in the case of disapproval), they tend to differ in the extent to which these attitudes are considered reasonable. Following Allport's (1954) classical definition, it has been argued that prejudice is antipathy based on a faulty generalization (e.g., Sampson, 1999). Prejudice would involve misconceptions, inaccuracies, and misjudgments based on personality dynamics, general cognitive errors, rigid forms of thinking, or unconscious biases. However, many social psychologists argue against this feature for defining prejudice because it is often very difficult, or even impossible, to establish the “incorrectness” or “veridicality” of prejudicial attitudes (e.g., Dixon, 2017; see also Jussim, Crawford, & Rubenstein, 2015). Furthermore, even when people may hold generally accurate stereotypes about groups, the application of these stereotypes to individuals still leads to errors and prejudices (e.g., Jussim, Cain, Crawford, Harber, & Cohen, 2009).

In addition to this “truth correspondence” problem, it is possible to understand prejudice in terms of what is considered socially reasonable and acceptable. It can be argued that “prejudice occurs when ‘we’ dislike ‘them’, and don't have a sensible reason for doing so” (Dixon & Levine, 2012, p. 10). This approach allows one to distinguish between intergroup negativity with or without sufficient warrant: group‐based negativity that does and does not seem reasonable and justified. It makes it possible, for example, to distinguish between the hostility that a violently subjugated population may feel toward their oppressors and the hostility that the latter feel toward the former (e.g., the antipathy of Jews toward Germans during the Nazi era, as opposed to the antipathy of Germans toward Jews; Duckitt, 1992). By simply operationalizing and measuring prejudice as a negative intergroup attitude, the differences between these sorts of situations disappear, and the possibility that negative reactions can be reasonably warranted is ignored (Dixon, 2017).

Obviously, what is considered reasonable depends on social, cultural, and historical circumstances. But this does not mean that there are no general moral principles and no shared social conventions about what is and what is not acceptable. The moral domain is typically concerned with fairness, justice, and others’ welfare and is considered to apply anywhere and everywhere (Graham et al., 2013; Haidt & Graham, 2007; Turiel, 2002). Research with children demonstrates that they interpret issues of fairness, justice, and avoiding harm to others as unalterable, general, and not subject to authority jurisdiction (see Wainryb, 2006). And research on moral emotions shows that people exhibit strong intuitive objections to the physical and psychological harm of others and unfair treatment (Turiel, Killen, & Helwig, 1987; but also see Haidt, Koller, & Dias, 1993), and that they tend to reject beliefs and practices that go against basic human capabilities (Turiel, 2002). It is, of course, not always clear whether a particular practice is interpreted as belonging to the moral domain and people argue about the interpretation and applicability of a moral principle, but typically not about the principle itself.

Treating categories of people (e.g., the elderly, the young, the sick) differently for relevant reasons (i.e., differential treatment) is something other than treating categories of people (women, African Americans, sexual minorities) differently on the basis of reasons that are considered irrelevant (i.e., discrimination). While there can be disagreements about whether particular reasons are relevant in a certain situation, a distinction between reasonable and just versus unreasonable and unjust is often codified in mores and legal rules and regulations (e.g., “beyond reasonable doubt”; “doctrine of reasonable classification”), and is essential in how people think about their social world. Without this distinction, almost all debate, decision‐making, and ruling would become impossible.

Disapproval tolerance is not based on outgroup fear or hatred, but involves objections that “must be reasonable in a minimal sense” (Cohen, 2004; Forst, 2013, p. 2). This means that the objections must not be arbitrary (e.g., use of double standards), unintelligible, and without moral value. It is generally more difficult to argue for and recognize the value and reasonableness of racist hatred and supremacy beliefs than of anti‐abortionists’ concern for the unborn life or the religious disapproval of secular beliefs and practices about euthanasia and gay marriage. People can think that there is nothing wrong with abortion, but still recognize that others may have reasons based on a commitment to a particular value for disapproving of it. And a Christian can be convinced that “Jesus is the only way” and disapprove of Hinduism as a belief system, while remaining egalitarian toward Hindus as a category of people. The prejudiced attitudes and discriminatory actions of a racist or bigot should not be equated with the reasonable concerns and critical remarks about other cultural and religious beliefs and practices (e.g., patriarchal, homophobic, misogynistic).

### 2.3 Behavior

Prejudice and disapproval also differ in the corresponding behaviors that they might predict. In a prejudice‐based approach, prejudice can lead to people either acting on their negative outgroup attitudes in the form of discrimination or restraining themselves or hiding their prejudicial attitudes in ways that prevents it from being openly expressed. Typically though, in such an approach, the negative attitude toward a group of people is in agreement with treating them, or wanting to treat them, in a negative way (i.e., you discriminate against those you dislike).

Some social psychologists argue that prejudice involves any attitude or behavior that implies outgroup antipathy (Brown, 2010). Forms of avoidance, exclusion, rejection, hostility, and discrimination are considered forms of prejudice, similar to negative beliefs and feelings. Others argue that negative behavioral intentions are the result of negative beliefs and feelings and these intentions would subsequently underlie negative behavior (Dovidio, Hewstone, Glick, & Esses, 2010; Jones, 1997). There is empirical support for these different theoretical models and each of them raises complex questions. However, one of the reasons for studying prejudice is that these would drive actual discrimination and other forms of negative behavior.

In support of this reasoning, there is a substantial literature on the relationship between attitude and behavior (e.g., Ajzen & Fishbein, 2005), and between prejudice and discrimination specifically (Schutz & Six, 1998; Talaska, Fiske, & Chaiken, 2008), indicating that people's behaviors (or intentions) are influenced by the attitudes they possess (Ajzen & Fishbein, 2005; Eagly & Chaiken, 1993). However, whereas some researchers find a close and direct relationship between the two, others find a weak or non‐significant relation (for a review, see Ajzen & Fishbein, 2005). Furthermore, the emotional dimension of prejudice is often more likely to drive discrimination than the cognitive dimension (Talaska et al., 2008), although this depends on the target group in question and on situational conditions (Esses, Haddock, & Zanna, 1993).The different findings are due to many factors including the level of specificity at which the attitude and the behavior are measured (Ajzen & Fishbein, 1977), the strength of the attitude (Howe & Krosnick, 2017), and whether people are conscious of their attitude and their negative behavior (Ajzen & Dasgupta, 2015). Importantly, behavior typically not only depends on attitudes, but also on individual goals, needs, and personality characteristics, as well as on social norms and opportunities (Ajzen, 2005; Conner & Armitage, 1998). Thus there are factors that can play an important role in preventing the translation of prejudicial attitudes into discriminatory action, which offers critical opportunities for prejudice‐reduction and anti‐discrimination interventions.

Disapproval can also lead to one of two outcomes. On the one hand, a person can maintain that the disapproved practice or belief is unacceptable and act intolerantly toward it by seeking to prevent it or to interfere with its expression. Alternately, one can determine that despite their disapproval for an outgroup belief or practice, there are good reasons (e.g., civic rights, religious freedom) to nonetheless tolerate the behavior by not interfering and enduring the beliefs and actions. Toleration, in this regard, implies putting up with specific beliefs and practices that you consider wrong (i.e., you accept what you disapprove of). It involves self‐restraint in order to prevent the negative attitude from becoming negative actions. It is, therefore, a barrier against discrimination and intergroup conflict. However, by doing so, toleration creates an inconsistency between one's attitude and behavior, thereby eliciting dissonance and uneasiness (Festinger, 1962; Harmon‐Jones & Mills, 1999). Such dissonance makes tolerance more demanding and difficult to maintain than intolerance in which the disapproval and rejection correspond (Gibson, 2006).

This potential of each negative attitude to lead either to interference, in the form of discrimination or intolerance, or non‐interference, in the form of prejudice suppression or tolerance, necessitates an analysis of the distinct psychological processes that underlie the prejudice‐reduction and toleration models, especially in regard to understanding what interventions might be successful and under which circumstances.

### 2.4 Psychological processes in prejudice‐reduction and toleration‐based approaches

**Figure 1:**
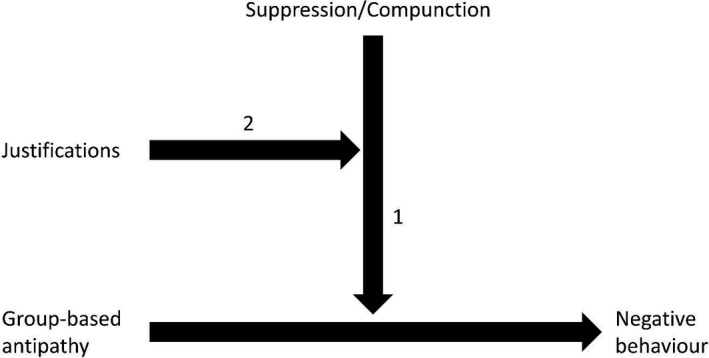
Psychological processes involved in a prejudice‐based approach

**Figure 2:**
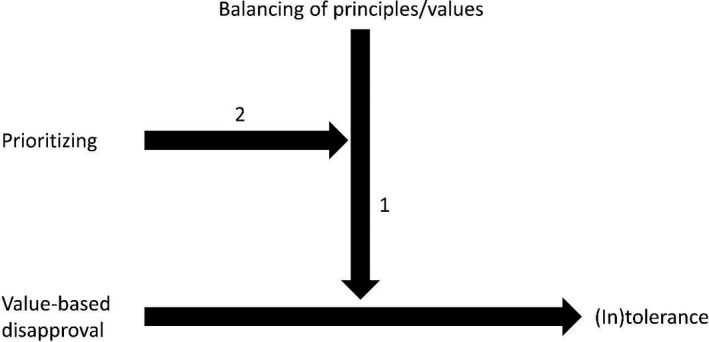
Psychological processes involved in a toleration‐based approach

We propose that underlying prejudice and disapproval are distinct sets of psychological factors that have important moderating effects for understanding and promoting the prejudice‐reduction and toleration‐based approaches. These factors play an important role in determining the consequences of the negative attitudes involved in prejudice and disapproval. Neither prejudice nor disapproval necessarily needs to translate into negative behavior. There are various reasons why people do not act upon their feelings of group‐based antipathy and there are various reasons why people tolerate things they disapprove of. Some of these reasons and the corresponding processes might overlap, but it is possible to identify different psychological processes and factors that give a further understanding of the differences between the prejudice‐reduction approach4There are various perspectives and approaches on trying to reduce prejudice, but here we focus on so‐called suppression models that are most similar to the tolerance‐based approach that is central to our discussion. and the toleration‐based approaches for improving intergroup relations, and how different interventions are likely to be needed to address these two sources of tension in diverse societies. These differences are visualized with the moderation pathways in Figures 1 and 2.

#### 2.4.1 Prejudice, suppression and compunction

When someone holds a prejudicial outgroup attitude, there are forces in play that may inhibit the expression of their negative feelings in verbal and non‐verbal behavior. Social psychological research has examined factors that moderate the release or suppression of group‐based antipathy. For example, work on aversive racism (Dovidio & Gaertner, 2004) argues that the desire to appear unprejudiced, together with the experience of outgroup discomfort and fear, leads people to discriminate only when their behavior is easily rationalized. Similarly, modern racism (McConahay, 1986) holds that people resolve the inner conflict between negative feelings about minorities and egalitarian beliefs by arguing, for example, that inequalities no longer exist. Allport (1954) claimed that prejudice with compunction is common because prejudicial attitudes often conflict with personally held values leading to inner conflicts and feelings of guilt and shame. The justification–suppression model suggests that people simultaneously hold negative outgroup beliefs, egalitarian values, and endorse social norms that suppress the expression of these negative beliefs (Crandall & Eshleman, 2003). These suppressors do not reduce the underlying group‐based antipathy, but rather inhibit its overt expression. Relatedly, the dissociation model of prejudice proposes that internalized anti‐prejudice normative and moral beliefs can override implicit negative stereotypes and feelings (e.g., Devine, 1989; Devine, Monteith, Zuwerink, & Elliot, 1991). Furthermore, the self‐regulation of prejudice model argues that people not only rationalize their prejudices, but also use normative and moral standards that make them motivated to control their prejudicial feelings at both the explicit and implicit levels (Monteith, Arthur, & McQueary Flynn, 2010; Plant & Devine, 1998).The desire to act in accordance with personal standards of egalitarianism (internal motivation to control prejudice) and the desire to avoid social punishments for expressing prejudice (external motivation to control prejudice) both play a role in the self‐regulation of negative intergroup behavior. These models differ in various respects, but all focus on internal regulation and control efforts to diminish the expression of group‐based antipathy.5A focus on people's motivation to respond without prejudice does not mean that the expression of prejudice cannot be intentional. Some people are motivated to express prejudice and tend to resist normative pressure to be non‐prejudiced (Forscher, Cox, Graetz, & Devine, 2015).


The efforts to regulate and control one's prejudices are typically based on the need to be viewed by oneself and others as unprejudiced so that one does not suffer internal (guilt) or external (shame) sanctions. People might accept that they are perhaps less competent or sociable than others, but they want to have a sense of being a morally good person and want to be recognized as such (Ellemers, 2017). Thus, the moderation line “1” in Figure 1 represents the regulating psychological forces that may inhibit the expression of group‐based antipathy with the related psychological conflict and turmoil.

#### 2.4.2 Prejudice and justification

While there are various conditions and factors that prevent people from expressing their prejudices, the group‐based antipathy involved in prejudice tends to “leak out” under certain conditions. Specifically, justifications (moderation line 2 in Figure 1) provide the opportunity to express prejudice without experiencing external or internal sanctions for doing so (Crandall & Eshleman, 2003). Justifications help individuals resolve the psychological conflict that derives from the need to be viewed as unprejudiced (by oneself and others) and the desire to engage in prejudiced behavior: with the proper justifications, people holding prejudicial attitudes tend to express their group‐based feelings of antipathy in biased acts.

The rationalization and expression of prejudice is facilitated by legitimizing myths that support unequal social arrangements (Sidanius & Pratto, 1999), status‐legitimizing worldviews (Major, Kaiser, O'Brien, & McCoy, 2007), conservatism (Sidanius, Pratto, & Bobo, 1996), negative stereotypes (Stephan & Stephan, 2000), threat perceptions (Pereira, Vala, & Leyens, 2009; Pereira, Vala, & Costa‐Lopes, 2010), perceived procedural and distributive justice (Louis, Duck, Terry, Schuller, & Lalonde, 2007), and processes of infra‐ and dehumanization (Haslam, 2006). For example, the availability of non‐racist justification facilitates discrimination by aversive racists (Gaertner & Dovidio, 1986). Further, “principled” conservatism is argued to cloak the underlying racism driving minority policy opposition (Sears & Henry, 2003; Sidanius et al., 1996; but see Reyna, Henry, Korfmacher, & Tucker, 2005).6There is a continuing debate about the “principles in principled conservatism”. On one side of the debate are theorists who argue that race‐neutral conservative values of equality, individualism, and fairness guide opposition toward specific minority policies (e.g., Sniderman & Tetlock, 1986), and on the other side are scholars who support a racism explanation of this opposition (e.g., Sears, 1988). Both sides have produced bodies of empirical research to support their interpretations and there is research that tries to reconcile these contradictory points of view (e.g., Reyna et al., 2005). Research also shows that processes of infra‐ and dehumanization alleviate moral concerns and thereby facilitate punishment and violence toward outgroups (see Haslam, 2006; Haslam & Loughnan, 2014). In a large‐scale study using representative samples of 21 European countries, it was found that when people perceive realistic and symbolic threats to the ingroup, they find it acceptable and justified to express opposition to immigration (Pereira et al., 2010). Similarly, in a series of eight studies, White and Crandall (2017) demonstrated that prejudiced people use the principle of free speech to justify someone else's anti‐Black behavior. Together, these different lines of research indicate that a motive for justification makes people look for (ideological) beliefs that legitimize their group‐based antipathy and its expression in negative outgroup behavior. Therefore, when seeking to engage in prejudice‐reduction and discrimination‐prevention, interventions can be modeled to increase suppression or compunction and also to challenge the justifications that people use.

### 2.5 Tolerance and psychological balancing

In contrast to prejudice, the value‐based disapproval of outgroup practices or beliefs is counterbalanced by other important values that the person holds that support tolerating the practices or beliefs despite their disapproval. The tolerance process implies a trade‐off between contrasting reasons for disapproval and for allowing the dissenting norms and practices: there need to be good reasons to permit behaviors or beliefs that trump the reasons for the disapproval not to be acted upon (see Figure 2, moderation path “1”). Toleration implies a dual form of thinking. On the one hand, there is what one sincerely believes is false or wrong, but on the other hand, one must be able and willing to allow others to live their life as they want. The psychological processes involved are not processes of suppression and compunction of feelings of outgroup antipathy, but rather the balancing between competing considerations.7This process is similar to what is proposed in the self‐regulation of prejudice model that we discussed earlier and which argues that people use normative and moral standards that make them internally and externally motivated to control their prejudicial feelings (Monteith et al., 2010; Plant & Devine, 1998). However, the starting point of this model is outgroup antipathy rather than reasonable disapproval of specific beliefs and practices, which makes the “input” of the balancing process of tolerance a question of weighting different moral reasons and concerns rather than a question of control efforts to diminish the expression of group‐based antipathy. People in a toleration‐based approach recognize different acceptable reasons for or against a specific action, weigh them up, and arrive at an all‐things‐considered judgement (Jones, 2010). Tolerating dissenting practices implies the belief that one is doing a good or right thing by doing nothing.

With toleration there are more important reasons for permitting than rejecting the disapproved beliefs and practices (Forst, 2013). For example, while a person's religious beliefs can commit them to view homosexuality as wrong, their civic egalitarian convictions can lead them to accept gay marriage and equal opportunities for gays and lesbians. Although there can be value‐based reasons for disapproval, it can be simultaneously emphasized that every citizen has an equal right to practice his or her culture or religion. The acceptance of the existence and equal rights of outgroup beliefs and practices (“respecting the right to hold particular differences”) is not necessarily the same as considering these beliefs and practices as being equally valid (“respecting the difference”): “respecting people is entirely compatible with thinking their views are wrong, confused, irrational, or wicked” (Crane, 2017, p. 181). In research among Tea Party supporters (Simon et al., 2018) and among Muslims living in Germany (Simon & Schaefer, 2016, 2018; Simon et al., 2018), it was found that respect for homosexuals and religious outgroup members (Muslims and atheists, respectively) as equal fellow citizens goes together with tolerance for those outgroups. Furthermore, respecting people as having intrinsic worth simply as a function of being persons has been found to be associated with stronger positive action tendencies and weaker negative action tendencies toward ethnic and religious (Muslim) minority outgroups (Lalljee, Tam, Hewstone, Laham, & Lee, 2009). Additionally, research has demonstrated that support for civic and democratic values is among the most important predictors of political tolerance (see Sullivan & Transue, 1999).

Majority group members in western nations might object to some dissenting beliefs and practices of Muslim immigrants because they consider conformity to norms and rules that govern civic relations critical for a cohesive and peaceful society (Parekh, 2000). Yet, maintaining established social rules and standards can be considered as less important than religious freedom and the right to follow one's own way. In the Netherlands, majority members who strongly take exception to the way in which they perceive Muslim immigrants as treating women and children overwhelmingly support the right of Muslim immigrants to maintain their cultural values and traditions (Sniderman & Hagendoorn, 2007; see also Hagendoorn & Poppe, 2012). This level of support was equal to those who had in every respect a favorable attitude toward Muslims. And in the German debate about the construction of minarets, some argue that minarets should be tolerated because of the commitment to freedom of religion or for reasons of peaceful coexistence (Schiffauer, 2013).

However, part of this process also allows for the conclusion to be intolerant of the specific outgroup belief or practice in question. Tolerance is not the ultimate good and a toleration approach presupposes the possibility of intolerance. The boundaries of tolerance are found where reasons for rejection of the objectionable behavior or beliefs remain stronger than the reasons for acceptance. The wrongfulness of pedophilia, honor killings, female genital mutilation, domestic violence, and forced marriage does not reside primarily in intolerance. Toleration of these practices implies culpable indulgence because it would infringe on the harm principle and the rights of others, which is typically considered more important than the freedom to live the life that one wants. In these cases, intolerance (or zero‐tolerance) is a positive rather than a negative response. Countries adopting multicultural policies (e.g., Sweden, Canada, the UK) do not accept every aspect of minority cultures and religions, but tend to apply the liberal minimum and the harm and rights principle to decide whether something should be accepted or not (Tawat, 2014). For example, in 2015, and as an amendment to several existing acts including the Immigration and Refugee Protection Act, the Canadian government introduced the “Zero tolerance for barbaric cultural practices act” intended to prevent polygamy, forced marriage, and honor killings. Similarly, Sweden was one of the first European countries to pass a Bill against female genital mutilation and to criminalize honor crimes. Toleration is not the same as relativism and sometimes people are at fault for tolerating what they should not (Kim & Wreen, 2003).

Psychologically, with intolerance there should be better reasons for rejection than for acceptance. For example, moralized entities and activities tend to lead to avoidance and rejection, rather than toleration (Rozin, 1999). People tend to be more tolerant toward actions that are based on a different factual view of the world (“they think it is like that”) and dissenting cultural traditions than on different moral beliefs (“they think that it is right and good”; e.g., Verkuyten & Slooter, 2007; Wainryb, 1993).When people view an issue as moral, they show greater discomfort with dissenting beliefs and do not tolerate it, regardless of who engages in it (Hirsch, Verkuyten, & Yogeeswaran, 2019; Wright, 2012; Wright, Cullum, & Schwab, 2008). For example, one can resist the idea of Muslims establishing an Islamic primary school, not because one feels negatively toward Muslims, but because one thinks that religion has no place in public education entirely (Dangubic, Stark, & Verkuyten, 2019). People can have liberal secular values that lead them to support bans on all religious symbols and not only minority religious symbols in the public sphere. There can be a more generic moral disapproval of the practice itself, independent of who is doing it (Bilodeau et al., 2018; Hurwitz & Mondak, 2002; Sniderman et al., 1989). Stronger moral conviction about contemporary societal issues was associated with lower political tolerance of those not sharing one's views, and also with lower intergroup tolerance (Skitka et al., 2013). Similarly, stronger perceived similarity in moral values of fairness and care was associated with higher outgroup tolerance (Obeid, Argo, & Ginges, 2017). Thus, reasonable intolerance is more likely if outgroup practices are perceived as causing harm to others (e.g., gender equality, child marriages) or as mistreating or threatening the freedom and civic rights of others (e.g., against ethnic minorities, or LGBT + rights).

#### 2.5.1 Prioritization

When there are acceptable reasons to disapprove a behavior, but also good reasons to accept it, the toleration approach requires that the competing principles and values be weighed against each other to determine the right course of action. Thus, the way people rank competing values (e.g., civil liberties or social order) plays an important role in tolerance judgments (Peffley, Knigge, & Hurwitz, 2001). For example, research has found that stronger endorsement of universalism predicts greater acceptance of Muslims’ civic rights and liberties whereas stronger endorsement of value orientations of tradition is related to lower acceptance (Van der Noll, 2014). This raises the psychological question of how people come to rank competing values and how this ranking affects tolerance judgments. Furthermore, individuals not only prioritize one value over another, but can also experience conflicts and dilemmas because contrasting or competing values may be simultaneously considered relevant and important. According to rhetorical psychology, thinking, at least implicitly, implies a debate about alternative viewpoints in which contradictory pairs of common‐sense phrases and maxims are central (Billig, 1987). Common sense contains contrary themes that provide the resources for thinking, and making judgements involves some consideration of counter‐arguments that also appear to be reasonable (Billig et al., 1988). Like discursive social psychologists (Edwards & Potter, 1992), rhetorical psychologists focus on discourse and investigate the ways in which contrary themes are situationally instantiated in ordinary talk.

From a cognitive perspective, whether a particular value guides one's actual judgment and behavior is not only dependent on the relative importance attached to it, but also on the situational cues that make that value salient and relevant (Fazio, 1986; Feather, 1990). Focusing on the effects of media framing on tolerance, Nelson et al. (1997) found that when news regarding political actions of the Ku Klux Klan was framed in terms of the importance of freedom of speech, participants had higher levels of political tolerance for this group compared to a situation in which the importance of public order was emphasized. These and other results (e.g., Nelson & Oxley, 1999; Vescio & Biernat, 2003; Zilli Ramirez & Verkuyten, 2011) suggest that when a particular value is both strongly endorsed and situationally salient, people tend to evaluate an event in terms of that value (Haider‐Markel & Joslyn, 2001; Zaller & Feldman, 1992).

The processes of determining which considerations take priority in the balancing decision to determine whether tolerance or intolerance is the best response are therefore key to understanding how disapproval and prejudice differ, as well as in determining the best way to promote tolerance. Whereas interventions seeking to reduce the disapproval or to suppress the moral reasons for disapproval are unlikely to succeed and may even backfire, interventions seeking to offer additional valued reasons to tolerate despite disapproval, or interventions seeking to prioritize reasons for toleration, are more likely to be successful at promoting toleration. In other words, toleration‐based approaches focus on improving intergroup relations in contexts where people disapprove of outgroup beliefs or practices for specific reasons. Such situations require a different approach from what we currently have in the prejudice‐reduction literature and may involve value prioritization, recognition of ideological dilemmas, and considering competing reasons to encourage toleration of outgroup beliefs, practices, or ways of living, in order to avoid intergroup conflict.

## 3 IMPLICATIONS FOR IMPROVING INTERGROUP RELATIONS

We have tried to argue that the prejudice‐reduction and toleration‐based approaches operate differently and call for different approaches to intervene and promote greater intergroup harmony in culturally diverse societies. Many current approaches addressing negative intergroup relations use prejudice reduction interventions that are grounded in varying theoretical perspectives such as personality dynamics, categorization processes, cognitive biases, societal norms, and intergroup dynamics. Strategies such as cooperative learning, intergroup contact, shared identity, perspective taking, and other approaches, have been proposed as interventions to improve intergroup relations. However, the empirical evidence for the effectiveness of these interventions is not always strong (see Aboud & Levy, 2000; Beelman & Heinemann, 2014; Paluck & Green, 2009) and they also can induce reactance and therefore stronger prejudice (e.g., Berndsen, Thomas, & Pedersen, 2018). In considering possible implications, our current aim is not to give an overview of this research or the various interventions that exist in the literature, but rather to consider the key differences between the prejudice‐reduction approach and a toleration‐based approach (Figures 1 and 2).

### 3.1 Addressing the attitude object

One primary difference between prejudice‐reduction and the toleration‐based approach is that the former tends to focus on categories of people, while the latter focuses on specific outgroup beliefs or practices. For example, in strategies that target categorization processes, the emphasis is on changing the categorization between “us” and “them” or using this categorization in a positive, multicultural way (Park & Judd, 2005; Verkuyten, 2014). Decategorization (Miller, 2002), recategorization (Dovidio, Gaertner, & Saguy, 2007), and cross‐categorization (Crisp & Hewstone, 2006) are strategies proposed for reducing prejudice. For example, with decategorization the emphasis is on the variability within an outgroup and the importance of seeing others as unique individuals rather than category members. Experimental research has indicated that decategorization can indeed lead to more positive outgroup attitudes (Ensari, Christian, Kuriyama, & Miller, 2012; Miller, 2002), and in a number of studies conducted in France it was demonstrated that increasing the perceived variability of minority outgroups (Moroccans, Arabs, Chinese) reduces prejudice and discrimination toward its members (Brauer & Er‐rafiy, 2011). And the strategy of recategorization makes an “us–them” distinction less salient by introducing a shared super ordinate category. A new, inclusive sense of “we” can ensure that the previous outgroup is incorporated and becomes one of “us” and thereby benefits from the preferences that usually exist for the ingroup. This does not have to imply that outgroup distinctiveness disappears because a dual identity or a multicultural model is possible in which separate group identities are affirmed within the context of a larger, inclusive whole (Dovidio, Gaertner, & Saguy, 2009; Dovidio et al., 2007; González & Brown, 2003; Hornsey & Hogg, 2000; Park & Judd, 2005).

However, these categorization approaches do not address the disapproval of specific beliefs and practices that is involved in intergroup toleration. The focus on human categories of prejudice‐reduction approaches can be limiting when it comes to finding solutions for intercultural tensions and conflicts about dissenting beliefs, practices, and worldviews, which are often moralized. Improving tolerance is not a question of minimizing category distinctions or celebrating category‐based diversity, but rather about enduring specific outgroup practices and beliefs that one continues to object to. Intergroup toleration inevitably implies an “us–them” distinction and is not about multicultural recognition. Similarly, while prejudice can be reduced by stimulating the perception of a shared category belonging (Dovidio et al., 2007), this perception does not necessarily have to reduce the disapproval of dissenting outgroup practices and beliefs (e.g., ritual slaughter of animals, gender inequality) that is based on one's own liberal or secular principles (Bilodeau et al., 2018; Imhoff & Recker, 2012). Toleration requires learning to accept the equal right of outgroup members to live the life that they want. With toleration, the intergroup categorization is salient and the focus is on enduring dissenting practices and beliefs. One cannot be expected to positively evaluate all outgroup beliefs and practices, especially when such beliefs and practices conflict with one's own values (e.g., atheists appreciating religious people's beliefs and practices, and vice versa). Therefore, a toleration‐based approach argues for learning that there are good reasons to “put up” with beliefs and practices that one continues to disapprove of.

### 3.2 Addressing attitude negativity

A related and critical difference between prejudice‐reduction and toleration‐based approach is that the former aims to change a negative attitude, while the latter does not. The various interventions for prejudice‐reduction aim to change people's group‐based feelings of antipathy. For example, taking the perspective of dissimilar others (for a review, see Todd & Galinsky, 2014), having positive contact with them (for a review, see Al Ramiah & Hewstone, 2013), and emphasizing a common identification with them (for a review, see Dovidio et al., 2009) can lead to less negativity toward the same group. Intergroup relations will improve if people experience and understand that their feelings of antipathy are unfounded and mistaken. So the aim is that prejudiced people will have fewer negative feelings and in turn give up their objections to, for example, the building of mosques and minarets, the wearing of a headscarf, religious education, education in minority languages, or same‐sex relationships.

However, these approaches do not address the source of toleration‐based processing. As disapproval is typically based on specific actions or beliefs of the outgroup, the group membership of the target does not have to play an important role in dictating the disapproval toward that action (but see Kuklinski, Riggle, Ottati, Schwarz, & Wyer, 1991). Thus, arguing for such a change in negative feelings is not arguing for toleration. A diversity that we appreciate and celebrate is not diversity in need of toleration. Tolerance is necessary when there is “deep” diversity in which one's own beliefs, values, and ways of life are considered to be in conflict with those of others. It enables us to accommodate others without giving up or diluting what we ourselves consider true and right. People will not give up their moral and religious values and beliefs, but rather try to fashion their life according to these and defend them when under attack (Brandt et al., 2014; Skitka, 2002; Tetlock, 2003). One's own cultural and religious beliefs and values provide the subjectively understandable and non‐arbitrary reasons for the disapproval. This does not have to imply narrow‐mindedness or a provincial outlook on the world, as illustrated by the famous quote of Mahatma Gandhi (1921): “I do not want my house to be walled in on all sides and my windows to be stuffed. I want the cultures of all lands to be blown about my house freely as possible. But I refuse to be blown off my feet by any” (p. 170).

### 3.3 Addressing behavior

Prejudicial feelings can be difficult to change (see Paluck & Green, 2009). Forms of self‐regulation and internal and external control are therefore important for making sure that prejudices do not result in discrimination and other negative behaviors. Social psychologists have examined inhibition processes such as banishing stereotypic thoughts from one's mind (Macrae, Bodenhausen, Milne, & Jetten, 1994), internal and external motivation to control prejudices (Plant & Devine, 1998), the role of diversity ideologies such as multiculturalism (Whitley & Webster, 2019), and social norms (Crandall, Eschelman, & O'Brien, 2002; Sechrist & Stangor, 2005). Although rebound effects, backlashes and moderating conditions have been documented (Berndsen et al., 2018; Danso, Sedlovskaya, & Suanda, 2007; Galinsky & Moskowitz, 2007; Morrison, Plaut, & Ybarra, 2010; Vorauer & Sasaki, 2011; Zhang & Hunt, 2008), such work has demonstrated that these factors can help prevent the expression of prejudice in negative behavior. Furthermore, forms of self‐regulation, self‐involvement, and social norms can change group‐based antipathy, not only at the conscious explicit level, but even at the implicit level (e.g., Crandall & Stangor, 2005; Lai et al., 2014; Paluck & Green, 2009). For example, the perception of what most others members of society think and feel (descriptive norms) or specific rules, regulations, and policies (injunctive norms) can change individual attitudes and behavior (Crandall & Stangor, 2005).

By contrast, toleration implies non‐interference with what one disapproves of, not out of indifference, fear, or feelings of threat, but rather because the reasons to endure and permit override the reasons to constrain, prevent, or forbid. Conflicting considerations are central to toleration, which means that it is not sufficient to tell people that they should give up their disapproval. Tolerance requires a standard, based on our beliefs and values, of what we think is best, together with establishing an allowable variation from that standard. In the absence of such a standard, one might find it easier to simply reject things that one disapproves of or rather try to take the seemingly moral high ground by just accepting almost everything. The implication is that toleration requires awareness and weighing of reasons to object to certain outgroup beliefs and practices with reasons to nevertheless accept them. Respect for others as autonomous persons and equal citizens is a particularly apt ground for tolerating beliefs and ways of life that we disapprove of (Schirmer, Weidenstedt, & Reich, 2012; Simon, 2007; van Quaquebeke, Henrich, & Eckloff, 2007).

A major challenge for prejudice‐reduction is that people can justify their group‐based antipathy and its expression so that they do not feel guilty or ashamed. As indicated in the moderation model of Figure 1, these justifications neutralize the psychological processes of self‐regulation, suppression, and social norms. The result is the open expression of prejudice and negative behavior. The implication for prejudice‐reduction is that these justifications should be targeted and challenged so that they lose their potency to provide cover for prejudice.

However, in the toleration‐based approach, intolerance is not always considered negative. Tolerance requires a standard of what we think is best, including when something should no longer be tolerated. Not everything can be tolerated and the reasons for not allowing dissenting practices and beliefs (e.g., harm and rights principle) can trump those for accepting these (e.g., religious freedom). This again means that in the toleration approach, the focus is on the nature of the different reasons and the weighting processes involved. In this approach, making people aware of and inducing them to think about the reasons for toleration and its boundaries is central (Jones, 2010).The fact that tolerance requires awareness of the dissenting practices and the various reasons is another difference from prejudice, which can be implicit and affect perception, evaluation, and behavior outside of awareness (Yogeeswaran, Devos, & Nash, 2016).

## 4 DISCUSSION

### 4.1 Theoretical implications

There is a tendency in the social psychological literature to consider all forms of (subtle) outgroup dislike and disapproval as forms of prejudice, all forms of justice‐based opposition as merely masking prejudice, and inaction as the result of suppressed antipathy that waits to be released when the right justification is there. This tendency implies a rather pessimistic and one‐sided perspective on human functioning and ignores the fact that for instance liberal and secular principles can be genuine determinants of opposition to specific minority practices (Sniderman & Hagendoorn, 2007) just as the fact that beliefs about sexual morality and the sanctity of life can underlie opposition toward abortion (Rodriguez & Ditto, 2019), and concern for procedural justice can be a genuine determinant of opposition to affirmative action (Bobocel, Son Hing, Davey, Stanley, & Zanna, 1998). Such a tendency misses out on understanding some of the principled and nuanced conditions involved in intergroup relations. The meaning of prejudice is broad and has been expanded over the years to include modern, aversive, symbolic, subtle, benevolent, and non‐conscious forms. There are good and valid reasons for doing so, but this conceptual broadening has ambivalent implications (Haslam, 2016). Theoretically, it becomes difficult to develop an understanding of those situations in which negative evaluations of groups (e.g., pedophiles, terrorists, extremists) are considered warranted and appropriate. And it also becomes difficult to consider those situations in which people disapprove of specific outgroup beliefs and practices (religious education, building of minarets, ritual slaughter of animals), but not of the outgroup as a category of people (e.g., Muslims, Jews). Disapproval can arise from principled commitments and basic values that have little to do with feelings toward specific social categories or groups.

The psychology of prejudice‐reduction and group‐based antipathy should be complemented with the psychology of practice‐based disapproval and tolerance. We have tried to argue that the negativity of disapproval differs from the antipathy of prejudice, the reasoning to tolerate differs from processes of prejudice, and the considerations behind intolerance differ from the justifications to express prejudice. A focus on tolerance advances the social psychology of intergroup relations and generates new directions for theory development and research (Jackman, 1977; Verkuyten & Yogeeswaran, 2017). It draws attention to the ways in which people try to evaluate and balance the different reasons for and against accepting specific outgroup beliefs and practices. Specifically, research on intergroup tolerance requires a close examination of the reasons (e.g., civil liberties) to nevertheless accept outgroup beliefs and practices that one continues to disapprove of. The psychological processes involved in these competing motivations and how these differ from self‐regulation processes in prejudice should also be examined. And it requires a focus on the reasons for the limits of tolerance (e.g., harms and rights principle) when outgroup beliefs and practices can no longer be tolerated.

It also requires a closer examination of the specific considerations people give to disapprove of others’ beliefs and practices, how these are similar to or different from feelings of antipathy, and when and why individuals object to outgroup beliefs and practices that are incompatible with their own without necessarily rejecting the category of outgroup people. Prejudice might make it more difficult to accept the equal rights of another group, and perceived social consequences of particular outgroup practices might fuel outgroup antipathy. Furthermore, perceptions of outgroup threat might make tolerance more difficult and processes of prejudice justification more likely. The political science literature has shown that perceived threat is one of the strongest predictors of reduced tolerance (Gibson, 2006) and social psychological research has demonstrated that threat increases prejudice (Rios, Sosa, & Osborn, 2018). In contrast, forms of positive intergroup contact might make people distinguish less between group‐based antipathy and disapproval‐based tolerance. Contact might not only lead to less prejudice (Al Ramiah & Hewstone, 2013) but also change the basis and strength of the disapproval (Chong, 1994; Ramos, Bennett, Massey, & Hewstone, 2019). People can get used to living around others with different cultural beliefs, customs, and practices, and can become more inured to things that once bothered them (e.g., abortion, gay marriage). This does not mean that they do no longer have objections, but these might be less strongly felt and thus involve less psychological tension and less need for balanced thinking and self‐restraint. Psychological adaptation means that people's feelings about the things that they tolerate can gradually change and the limits of their tolerance can alter.

Toleration implies an asymmetrical relationship because we can only tolerate what we can prohibit (Cohen, 2004). Individuals from one social group (e.g., majority members) are in the position to interfere with the way of life of individuals from another group (e.g., minority members). This means that intergroup tolerance inevitably raises questions of status and power, whereby the majority conditionally permits dissenting minority groups to live according to their way of life. The qualified permission to minority group members to live according to their beliefs affirms the dominant position of the majority and the dependent position of the minority. Therefore, some critics argue that tolerance functions as a subtle social mechanism contributing to domination and inequality (Insel, 2019; Marcuse, 1965), and to the depoliticization of diversity by reducing structural disadvantages to cultural group frictions (Brown, 2006). The theoretical implication is that tolerance should not only be studied in terms of meaningful differences in moral values and beliefs, as we have done here, but also in terms of intergroup processes, as we have discussed more fully elsewhere (Verkuyten & Yogeeswaran, 2017). For example, the notion of tolerance is not only useful to argue for the acceptance of minority group practices and beliefs, but also for construing an ingroup‐favoring moral distinction between “us” and “them”. As frequently argued in debates about immigration and diversity, western societies would coalesce around core values of equality, freedom, and tolerance. This argument is typically made in comparison to the alleged intolerance of some immigrant groups, and Muslims in particular (Verkuyten, 2013). Furthermore, toleration often is inescapably patronizing and condescending, and can be considered offensive and hurtful by those who are tolerated because it implies disapproval of what they believe and practice (Verkuyten et al., 2019). However, there has hardly been any empirical attention to the psychological implications of being the target of toleration and whether this has a negative impact on, for example, minorities’ well‐being and collective action tendencies (but see Cvetkovska, Verkuyten, & Adelman, 2019).

### 4.2 Applied implications

Not all forms of dislike and disapproval can be productively understood within the prejudice‐reduction approach. Doing so is not only theoretically limiting but also makes it difficult to develop a detailed and nuanced understanding of people's considerations in trying to deal with their everyday multicultural concerns and dilemmas. It becomes very difficult to change people's views when their concerns about cultural diversity or their negative experiences with cultural and religious others are disqualified and dismissed as being racist (“mad and bad”). For changing their views, we need to understand how they come to think negatively of others with, according to them, sufficient warrant. For example, in her extensive fieldwork among Tea Party supporters in the US, Hochschild (2016) identifies a “deep story” in which people feel betrayed in their country: “strangers step ahead of you in line, making you anxious, resentful and afraid. A president allies with the line cutters, making you feel distrustful, betrayed. A person ahead of you in line insults you as an ignorant redneck, making you feel humiliated and mad” (Hochschild, 2016, p. 222). People want to be morally good persons, but they are also morally outraged when they feel betrayed and when their genuine concerns about immigration and cultural diversity are not taken seriously, but rather reduced to prejudice and racism (Gest, 2016; Verkuyten, 1997).

Just as prejudice‐reduction should not be a substitute for tolerance, toleration should not replace prejudice‐reduction. Prejudiced people should not learn to endure the outgroup they dislike, but rather change their group‐based antipathy and hatred, and overcome their racist beliefs. The right reaction to a bigot or racist is not to ask him or her to be tolerant, but rather to change his or her prejudices and racism (Crick, 1971; Forst, 2013). Generalized outgroup negativity and beliefs about outgroup inferiority should be addressed for what they are. Furthermore, prejudice‐reduction approaches might be important for successful toleration interventions because toleration requires respect for the autonomous and equal (citizen, human) status of cultural and religious others (van Quaquebeke et al., 2007; Simon, 2007). In contrast, tolerance interventions may not reduce prejudice because tolerance draws attention to equal rights for outgroup practices and beliefs rather than to categories of outgroup people. For example, teaching middle school students about the norms and principles of democracy was found to enhance their political tolerance of relevant outgroups, but it also made their dislike of the groups in question stronger (Avery, Bird, Johnstone, Sullivan, & Thalhammer, 1992).

In contrast to research on prejudice‐reduction, there is very little systematic research on the effectiveness of toleration‐based approaches for addressing intergroup tensions and conflicts. Toleration is far from easy because it requires the weighing of reasons to object to certain outgroup beliefs and practices with reasons to nevertheless accept them. This implies that people should be made aware of and sensitive to the reasons for toleration and its boundaries. Thinking about particular examples or prototypes of tolerance might be important for learning to make judgments about issues of tolerance (Sniderman et al., 1989). For example, thinking about civil liberties and the importance of letting people say what is on their mind might form paradigmatic examples of tolerance. Thus, discussing relatively straightforward cases is probably a good starting point for teaching youth and ordinary citizens the importance of tolerance (Jones, 2010). There are relatively uncontroversial cases of intolerable conduct and there are many examples in which intolerance is inappropriate because the disapproval is hard to defend. These can form the impetus for evaluating more complex real‐world issues, although there is always the possibility that thinking about these issues and their consequences leads people to consider a range of other principles and values (e.g., social order, tradition) that reduce their tolerance (Kuklinski et al., 1991).

There are cultural and religious differences about what is right and wrong, just and unfair, and how we should relate to one another. Living with diversity inevitably creates situations where we are faced with outgroup beliefs or practices we disapprove of and trying to remove such disapproval may not be easy, or even possible (e.g., trying to persuade liberal activists to appreciate conservative speakers on campus, or trying to persuade religious believers to appreciate atheism). It is about these differences that ways of life collide and toleration becomes a necessity for pluralistic societies. Toleration is a minimal condition for living together despite meaningful differences (Vogt, 1997). It is a barrier against discrimination, hostility, conflict, and a critical condition for citizenship and democracy (Sullivan & Transue, 1999). Toleration provides access to resources and rights, and allows cultural and religious minority group members (to a certain extent) to live the life that they want. However, tolerance is not the only or ultimate goal for intergroup relations in pluralistic societies, and stimulating toleration should not replace the promotion of mutual recognition between groups and substantive forms of inclusion.

## 5 CONCLUSION

There is a large and important literature on the social psychology of prejudice. Much is known about the nature, causes, and consequences of prejudicial attitudes, and of ways to reduce prejudice. Modifying prejudice is without doubt very relevant and critically important for intergroup relations in pluralistic societies because it reduces the risk of forms of negative outgroup behavior and intergroup conflict. But in our view, the relevance of social psychology for the general problem of multicultural coexistence can and should be broadened by a concern with intergroup tolerance (Jackman, 1977; Verkuyten & Yogeeswaran, 2017). The disagreements and tensions arising from meaningful cultural and religious diversity need a focus on toleration in many settings. Tolerance has become a buzzword in national, international, and organizational and institutional settings for establishing multicultural justice and peaceful coexistence (Brown, 2006). But how toleration exactly differs from prejudice‐reduction is often not clear and there is little social psychological research on intergroup toleration. We have tried to show that the theoretical and practical questions raised by the toleration approach differ from those raised by a prejudice‐reduction approach. In our view, systematic attention to questions of tolerance can enhance psychology's contribution to the development of positive intergroup relations by stimulating theory development and raising novel questions for empirical research.

## CONFLICT OF INTEREST

The authors declare that there are no potential conflicts of interest with respect to the authorship and/or publication of this article.

## ETHICAL STATEMENT

This is a theoretical article and there were no ethical concerns related to empirical research.

## TRANSPARENCY STATEMENT

No research material and data were analysed in this article.
